# Laparoscopic Splenopexy Due to Wandering Spleen: Feasible Technique

**DOI:** 10.7759/cureus.22597

**Published:** 2022-02-25

**Authors:** Fatih Sumer, Gokalp Okut, Kuntay Kaplan, Orgun Gunes, Cuneyt Kayaalp

**Affiliations:** 1 Gastrointestinal Surgery, Irmet Hospital International, Tekirdag, TUR; 2 Gastrointestinal Surgery, University of Health Sciences Izmir Bozyaka Training and Research Hospital, Izmir, TUR; 3 Gastrointestinal Surgery, Adana City Hospital, Adana, TUR; 4 Gastrointestinal Surgery, Izmir Katip Celebi University Faculty of Medicine, Izmir, TUR; 5 Gastrointestinal Surgery, Yeditepe University Medical School, Istanbul, TUR

**Keywords:** non-absorbable mesh, detorsion, wandering spleen, minimally invasive laparoscopy, laparoscopic splenectomy

## Abstract

Wandering spleen (WS) is a rare disease caused by the looseness of the splenic ligaments. A 29-year-old female patient presented to the emergency department with complaints of abdominal pain and a palpable mass in the abdomen. A diagnosis of WS was made as a result of preoperative imaging. We performed urgent laparoscopic splenopexy with non-absorbable mesh in a patient with torsioned WS. WS is a disease that must be operated on urgently because it causes ischemia and necrosis in cases where it causes torsion in the splenic pedicle. Many researchers also recommend surgery in asymptomatic patients. While splenectomy was previously recommended for WS, current recommendations advocate for splenopexy. As a result, the only and definite treatment option in the case of WS is surgery. Splenopexy with minimally invasive techniques should be the first choice if possible. Splenopexy with non-absorbable mesh is an inexpensive and feasible method to prevent re-torsion. The use of non-absorbable mesh in laparoscopic splenopexy has not been shared before in the literature.

## Introduction

Wandering spleen (WS) is a rare condition and was first described on an autopsy in 1667 by a Dutch doctor, Van Horne. In this disease, there is laxity or absence of splenic ligaments. Apart from its normal position, the spleen can be found anywhere in the abdomen, from the bottom of the left diaphragm to the pelvis [1]. There is a risk of pedicle torsion and splenic infarction in this case. Patients often require splenectomy for WS, but it is now reserved for those with non-viable spleens. Splenopexy should be the first choice in viable spleens [2]. For these reasons, many splenopexy techniques have been defined and performed. This study presents a case where we performed urgent laparoscopic splenopexy with non-absorbable mesh in a patient with torsioned WS.

## Case presentation

**Figure 1:**
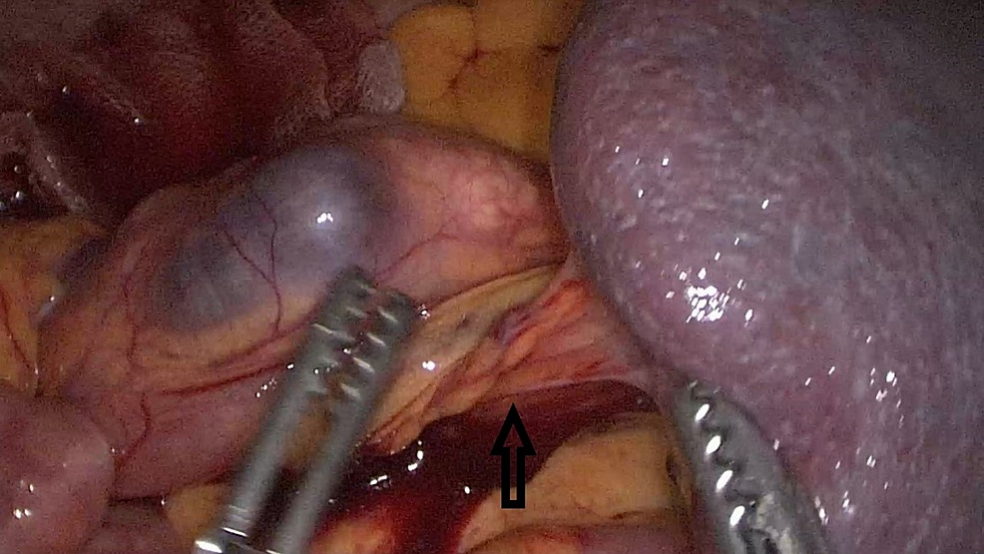
Torsioned splenic hilum (Black arrow)

**Figure 2:**
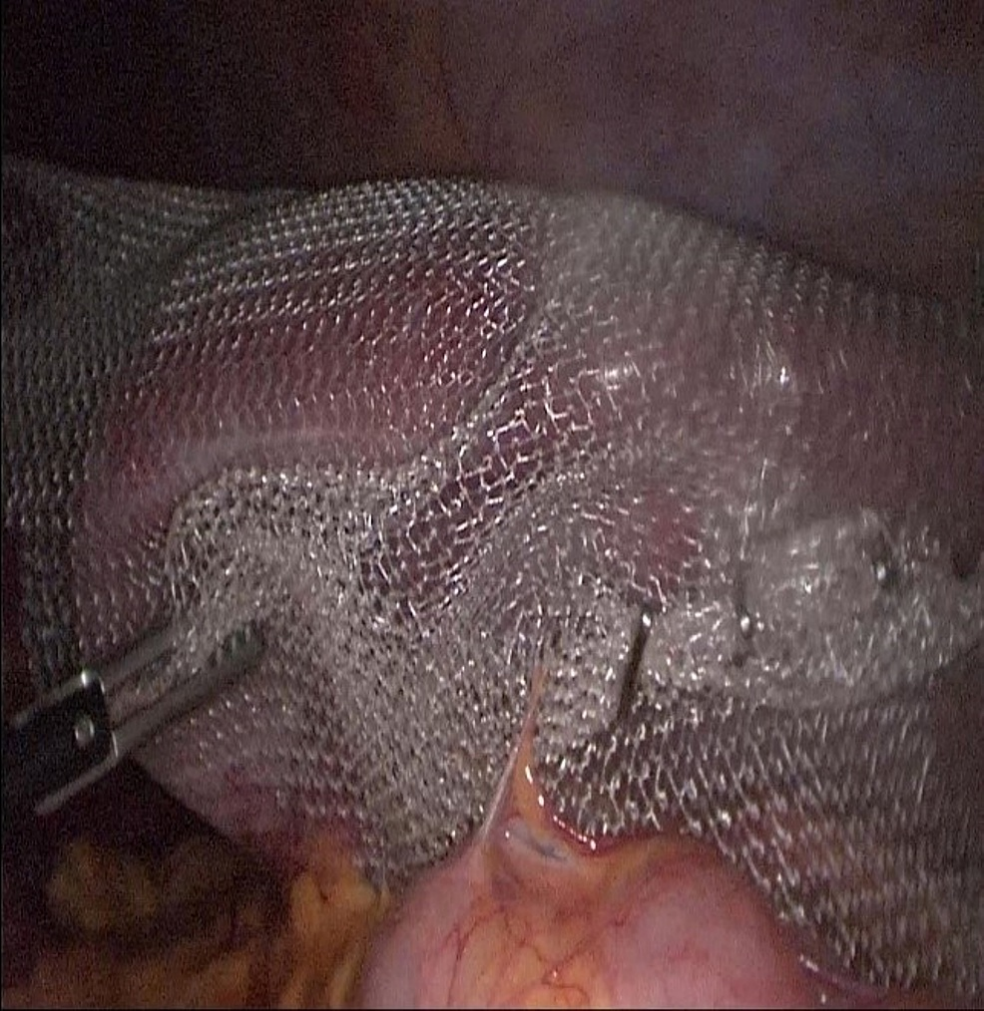
Suspension of the spleen in the left upper quadrant

**Figure 3:**
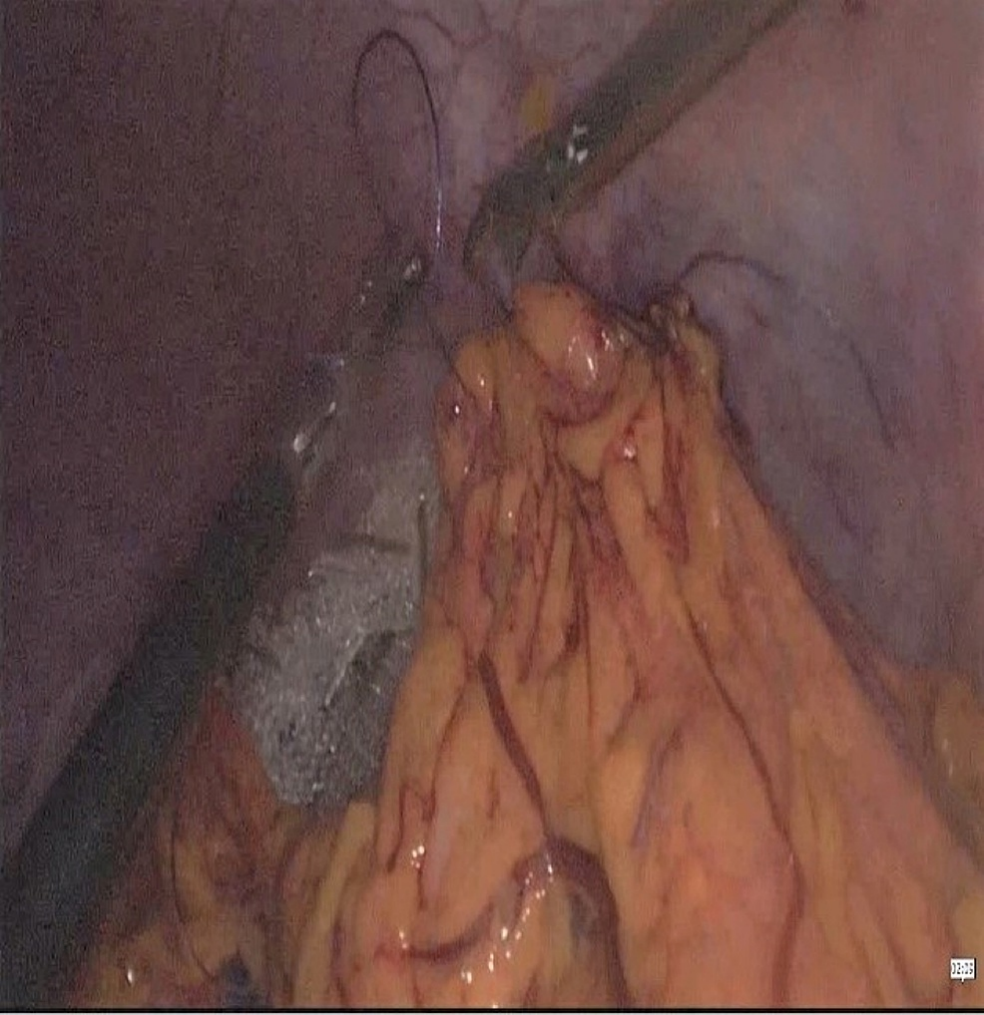
Wrapping the prolene mesh with the omentum

A 29-year-old female patient presented to the emergency department with complaints of abdominal pain and a palpable mass in the abdomen. Her past medical history was unremarkable. There was a 20*15 cm mass extending from the umbilicus to the right lower quadrant on physical examination. Body mass index: 17.3 kg/m². The patient's blood tests were normal. Abdominal ultrasound revealed the absence of spleen in the left upper quadrant and a mass compatible with a 22*18 cm spleen in the right pelvis. Triphasic abdominal tomography revealed a mass compatible with the spleen in the pelvis, 720° torsion in the splenic vein, and free fluid in the lower abdominal quadrants, and there was blood flow in the splenic artery. WS and related torsion were considered. A single dose of prophylactic antibiotic was given before surgery. During the operation, the patient was in the supine position, the surgeon and the camera assistant were placed on the right side of the patient, with the monitor on the left side of the patient, 10mm trochar inferior umbilicus, 10mm trochar from the right midclavicular line level, from the lateral umbilicus and 5mm trochar from the right upper quadrant. It was observed that the spleen was located in the pelvis together with the distal pancreas, and there were two torsions in the pedicle. It was found that the spleen was viable and larger than normal. It was observed that the spleen dimensions returned to normal after being detorsion (Figure 1). The spleen was repositioned. After packing with prolene mesh (Figure 2), it was fixed with a tack fixation device. The omentum was fixed in the abdominal wall with 3/0 vicryl to isolate the mesh from the abdomen (Figure 3). The operation time was 180 minutes, and the bleeding was 50cc. Postoperative Doppler ultrasonography and abdominal tomography revealed the spleen in normal localization, and splenic vascular inflow and outflow were patents. The patient was discharged on the fifth postoperative day. No problem was observed in the patient in the fifth year of follow-up.

## Discussion

Although WS is most common in the neonatal period (primarily in men), it is seen ten times more in women than men after the age of ten [3]. While adults usually present with symptoms of an abdominal mass, children generally present with abdominal pain [4]. Some patients may present with more vague symptoms such as nausea, vomiting, early satiety, cramping abdominal pain. Apart from these, more rare symptoms such as gastric volvulus, gastric outlet obstruction, pancreatitis, celiac trunk obstruction, small bowel obstruction, and spleen rupture have been reported [5-7]. In these cases, it is often difficult to diagnose without radiological assistance. Our case presented with a mass in the abdomen, partial venous return disorder in the spleen, and abdominal pain, which we think is venous congestion. It was thought that the patient might have WS, and the diagnosis was confirmed by abdominal ultrasonography and tomography.

In symptomatic patients, splenopexy can be performed if the spleen is viable, but splenectomy should be performed if there is necrosis in the spleen. Both splenectomy and splenopexy can be performed laparoscopically [7]. In asymptomatic patients, many researchers recommend surgery in terms of future symptoms and complications [8]. One study found the rate of developing complications in asymptomatic patients as 65% [9]. Many peritoneal and extraperitoneal techniques have been described for splenopexy. These include splenopexy with intraabdominal absorbable mesh, splenopexy by creating an omental pocket, wrapping the spleen to the left upper quadrant by wrapping it with a double-leaf mesh called the sandwich technique, and splenopexy in which the spleen is placed in this pocket by creating a retroperitoneal pocket. Due to the rarity of the disease, it is impossible to compare the superiority of these techniques with a prospective randomized study [4,6,8]. In our case, the spleen was found to be viable. Spleen size returned to normal after detorsion. Since there was no absorbable mesh in our center, the spleen was packed between the two sheets of prolene mesh using the sandwich technique and then fixed to the left upper quadrant. Omentopexy was performed to cover the anterior abdominal wall, covering the spleen completely.

Splenectomy is required in 50% of patients with acute ischemia with torsion due to WS [10]. Therefore, the patient diagnosed with torsioned WS should be operated on immediately. After the spleen is detorsion, it should be decided whether it is viable or not. While splenectomy was used as a standard in the past, laparoscopic splenopexy has become the gold standard in patients where possible.

## Conclusions

As a result, the only and definite treatment option in the case of WS is surgery. The first and most important step in diagnosis is to suspect this disease. Splenopexy with minimally invasive techniques should be the first choice in possible patients. Splenopexy with non-absorbable mesh is an inexpensive and easily applicable method to prevent re-torsion.
